# Contagion beyond the virus: A case obsessive-compulsive disorder centered on Covid-19

**DOI:** 10.1192/j.eurpsy.2024.1037

**Published:** 2024-08-27

**Authors:** A. S. Pires, M. Pires, J. Nunes, I. F. Vaz, S. Mouta, B. Jesus, D. Cruz e Sousa, D. Figueiredo

**Affiliations:** Department of Psychiatry and Mental Health, Local Health Unit, Guarda, Portugal

## Abstract

**Introduction:**

The Covid-19 pandemic has generated an unprecedented impact on multiple levels (health, occupational, economic, and social) which affected the general population and has been an enormous stress factor for individuals with obsessive-compulsive disorder (OCD), particularly for those with contamination symptoms. Many patients, as well as healthy individuals, experienced new obsessive-compulsive-like symptoms centered on COVID-19 during the pandemic. However, data on this population are still scarce.

**Objectives:**

To present a case exemplifying the association between the Covid-19 pandemic and the onset of OCD.

**Methods:**

Case presentation and non-systematic review of existing literature on Pubmed using the keywords: Covid-19, OCD, pandemic, depression.

**Results:**

We report a case of a 30-year-old female who presented to the emergency department due to depressive mood and suicidal ideation associated with exacerbation of OCD symptoms, namely intense fear of being infected with Covid-19. These symptoms led to avoidance of touching objects, surfaces or even herself in addition to frequent and long rituals of hand-washing and showers. She was asymptomatic prior to being infected with Covid-19, when she started developing obsessive ideas of contamination. She sought psychiatric support and was medicated with fluoxetine, olanzapine and clonazepam. Due to insufficient symptom control, she was admitted to the psychiatry ward, where treatment was initiated with aripiprazol and fluvoxamine. After dose titration, gradual remission of OCD symptomatology and depressive mood was observed.

**Conclusions:**

The present case illustrates the correlation between Covid-19 and the onset of OCD symptomatology. Existing studies demonstrate that the pandemic worsened the landscape of symptoms of OCD, both in diagnosed patients as well as in previously healthy individuals. However literature is still limited thus, multinational and cross-cultural, longitudinal studies are warranted to gain further insights on this topic.

**Disclosure of Interest:**

None Declared

